# Male breast cancer: clinicopathological characterization of a National Danish cohort 1980–2009

**DOI:** 10.1007/s12282-020-01066-3

**Published:** 2020-02-27

**Authors:** Anne Marie Bak Jylling, Vibeke Jensen, Giedrius Lelkaitis, Peer Christiansen, Sarah Schulz Nielsen, Marianne Djernes Lautrup

**Affiliations:** 1 Research Unit of Pathology, Department of Clinical Research, University of Southern Denmark, Odense, Denmark; 2 Department of Pathology, Odense University Hospital, Odense, Denmark; 3 Department of Pathology, Aarhus University Hospital, Aarhus, Denmark; 4 Department of Pathology, Rigshospitalet, Copenhagen University Hospital, Copenhagen, Denmark; 5 Department of Plastic and Breast Surgery, Aarhus University Hospital, Aarhus, Denmark; 6 Danish Breast Cancer Group, Rigshospitalet, Copenhagen University Hospital, Copenhagen, Denmark; 7 Department of Surgery, Lillebaelt Hospital, Vejle, Denmark

**Keywords:** Male breast cancer, Pathology, Clinicopathological characterization, Intrinsic subtypes

## Abstract

**Background:**

To describe relevant pathological parameters of Danish male breast cancer patients (MBCP) diagnosed from 1980 to 2009, and to relate these data to treatment, overall survival (OS) and standardized mortality rate (SMR).

**Materials and methods:**

The MBCP cohort was defined from national Danish registers. A total of 643 MBCP were identified with tissue available in 457. Among these, 384 were primary operable. Where tissue blocks were available, tumor type, grade, estrogen receptor (ER), progesteron receptor (PgR) and androgen-receptor (AR) status as well as HER 2 and Ki67 were performed. OS was quantified by Kaplan–Meier estimates and SMR was calculated based on mortality rate among patients relative to the mortality rate in the general population.

**Results:**

Male breast cancer was more often of ductal type, grade II and a very high proportion were ER and AR positive and HER2 negative. Intrinsic subtypes based on immunohistochemical evaluation showed luminal subtype. Ki67 ratio increased over period of study. OS declined by increased age, bigger tumor size, positive lymph node status, higher grade and Luminal B subtype. Hazard ratio and relative risk of SMR were highest for patients aged < 60 years.

**Conclusion:**

Male breast cancer is of luminal subtype, but more often Luminal B. Ki67 is crucial in evaluation of subtypes by immunohistochemistry, but have limitations. Subtyping seems to be of major importance. AR also can have a role in future treatment.

**Electronic supplementary material:**

The online version of this article (10.1007/s12282-020-01066-3) contains supplementary material, which is available to authorized users.

## Introduction

Male breast cancer represents less than 1% of all new cases of breast cancer. The incidence is shown to be increasing in certain studies as well as documented in the National Cancer Institute’s Surveillance, Epidemiology, and End Results Program (SEER) cancer statistics review (1975–2011) and NORDCAN [1–3]. Men with breast cancer are generally older than female patients [4].

The risk of male breast cancer increases with age [5].

Most former studies report worse outcome for male breast cancer than for female breast cancer based on overall survival. This seems to be correlated to older age at diagnosis, men have shorter expectation of life than women, comorbidity, later diagnosis and more advanced stage [1]. Because of that SMR seems to give a more relevant information.

In general, it is more difficult to do science on a rare disease like MBC because of small study populations and incomplete data because of long study periods including old data. Furthermore, different statistical methods for estimating prognosis or survival have been used.

Male breast cancer is often reported to be diagnosed at a later stage than female breast cancer, and differences in tumor biology have also been described [6, 7]. Male breast cancer is associated with BRCA2 gene [2, 5, 8] and more men have other malignancies [9, 10]. MBC is more often of ductal origin compared to female breast cancer and is almost always ER positive [11, 12]. Studies made on intrinsic subtypes based on histopathological criteria show that almost all are of luminal subtype and most often Luminal A compared to Luminal B, although results are conflicting [11, 13, 14]. Only very few and small studies doing molecular subtype, based on PAM50, showed Luminal B to be more common [15].

Much effort is made through translational research for showing that clinical, biological, pathologic and genetic parameters not only could be used for generating prognosis estimates, but also could predict the effect of a given form of treatment. Examples of such parameters in breast cancer include ER and endocrine treatment as well as overexpression/amplification of the HER2 receptor and HER2 targeted treatment. Because of small studies and lack of data, men are usually treated in the same way as female breast cancer patients, however, there are no clear recommendations in this area.

## Objective

The purpose of this study is to describe the prognostic and predictive biological and pathological markers, based on examination of collected paraffin-preserved tumor material from a large Danish cohort of male breast cancers in the period from 1980 to 2009.

Beyond that, the aim is to be able to identify areas where it will be relevant to test new, targeted regimes of treatment in prospective studies that may follow.

## Patients and methods

### Study population and period

The patient material was identified from the Danish Cancer Registry for the period 1st January 1980 through 31st December 2009—the same cohort as in a formerly published article presenting clinical data by the same authors [6].

To avoid misclassification, we double checked with two other Danish national registers; The National Patient Register (NPR), and The National Pathology Data Bank (Patobank).

Until now, men were not registered in the Danish Breast Cancer Group (DBCG) database, which only includes female breast cancer patients.

In Denmark, every person is registered with a civil registration number (CPR number) indicating time of birth. This allows for easy identification in any register of the total cohort of male breast cancer patients during the study period. Date of death is registered in the Danish Civil Registration Number System (CPR Register), too. We use the CPR number to link the different registers.

This means that we have complete data according to study population.

This information was used in the survival analyses.

Denmark has a tax-supported public health system providing free hospital care, and all patients treated in a hospital are registered in NPR with a code of diagnosis and a code of treatment supplied with a code of the hospital and the department treating the patient, allowing for identification of the location of the patients´ medical forms.

All pathology data from breast cancer patients are registered in Patobank. Patobank contains information in a database (or, for the very early period, in paper form) of all tumor pathology characteristics and lymph node involvement, reported at time of diagnosis.

For each patient identified through the registers, medical records were reviewed region-by-region with the aim to collect data on age, diagnosis (mammography including ultrasonography, clinical examination and biopsies, i.e., triple test), and treatment (surgery—radical/not radical, adjuvant chemotherapy, endocrine therapy and radiation therapy). Based on the available information from the registers and from medical forms, the cases of male breast cancer were classified as early, locally advanced, or disseminated at time of diagnosis. Furthermore, we evaluated from the medical forms if adjuvant therapy was considered sufficient and relevant according to existing guidelines for female patients.

Patients with disseminated disease and locally advanced disease at diagnosis were excluded if they never reached operation and thus, not enough tumor tissue was available.

In such cases, paraffin-embedded tumor tissue was requested from all Danish Departments of Pathology. In some cases, tissue blocks were not available. As expected, the frequency of unavailable tissue samples was higher for the early calendar periods.

Tumor tissue was handled at Department of Pathology, Aarhus University Hospital and all formalin-fixed, paraffin-embedded tissue blocks from the primary tumors underwent central pathology review for tumor type and grade, and new immunohistochemical analyses (ICH) were performed.

**Fig. 1:**
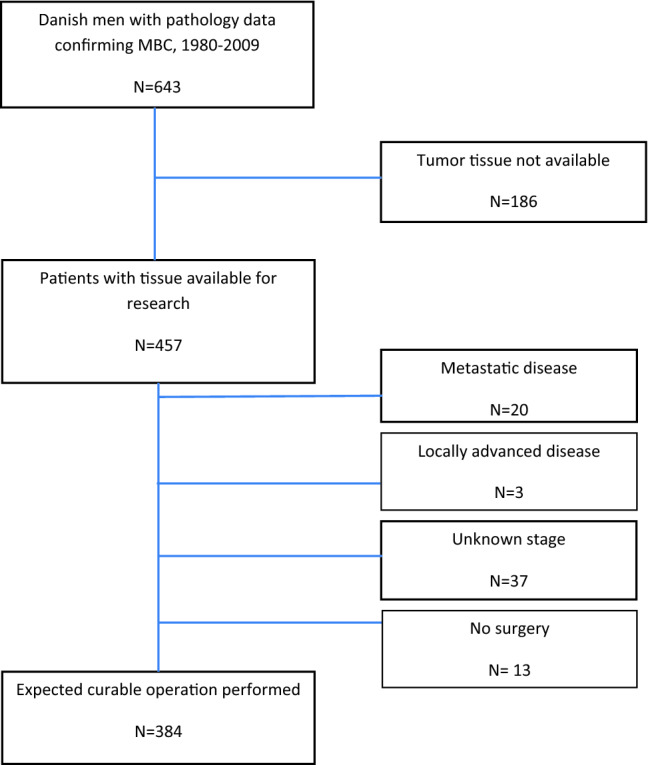
Danish male breast cancer population. Flow diagram

Patients with non-invasive breast cancer, misclassified breast cancer (for instance, metastases from other primary sites), those treated with neo-adjuvant therapy and patients with no or insufficient tumor tissue left were excluded. Furthermore, tumor tissue from all patients diagnosed at private practitioners or departments, which no longer exist, were not available. We were able to collect tumor tissue from 457 out of 643 patients with confirmed diagnosis during the period 1980–2009 and who were alive at diagnosis. 384 of these were considered having early-stage breast cancer and thereby primarily operable (Fig. 1).

### TMA preparation and evaluation

Since 1980, breast cancer treatment has gradually been centralized, and a lot of departments previously treating breast cancer are now closed. Therefore, some of the male breast cancer patients could not be localized and their medical records were not available. These patients remained included, but with missing variables. As expected, the frequency of missing medical records was higher for the early calendar periods.

The most representative tumor block (if more than one) was selected for the study. Whole slides were stained with Hematoxylin and Eosin (HE) and reviewed by three experienced breast pathologists for histological type and malignancy grade according to the modified Bloom–Richardson score [16, 17]. This slide was also used to identify representative, invasive tumor areas.

For the IHC analyses, tissue microarrays with two cores of 2 mm per tissue block from this area were obtained and embedded in a recipient paraffin block. Histological sections were cut at 4 μm and mounted on Superfrost+ glass slides and stained and scored for ER, PR, AR, HER2 and Ki67. All stains were performed on a fully automated IHC staining machine (BenchMark Ultra, Ventana). Appropriate positive controls were used throughout. HER2 2+ tumors were evaluated using Silver in Situ Hybridization (SISH). All IHC stains were performed in one center and scored in consensus by the same three breast pathologists, to prevent inter-laboratory and inter-observer variability and to assure quality of the data. We found this specifically important for the evaluation of KI67.

Results were considered positive if more than 10% of the cells were labeled for all markers, except Ki67: low if < 14% and high if ≥ 14% according to St. Gallen guidelines of 2013 [18]. HER2 membrane staining was assessed according to the national DBCG guidelines published in 2013 with the following recommendations of HER2 in breast cancer: negative if 0/1+ and positive if 2+, ISH confirmed or 3+.

Intrinsic subtypes were classified according to immunohistochemical panel expression profile as follows:

- Luminal A: ER+ and/or PR+, HER2 negative and Ki67 < 14%
- Luminal B: ER+ and/or PR+, HER2 negative and Ki67 ≥ 14%
  - ER+ and PR neg, HER2 negative and any Ki67
  - ER+, any PR, HER2 positive, any Ki67
- HER2 enriched: ER− and PR− and HER2 positive
- Triple negative: ER− and PR− and HER2 negative

### Statistics

Associations between characteristics were analyzed by Chi-square of Fischer’s exact test.

OS was calculated as the time elapsed from the date of surgery until death from any cause, and were estimated using the Kaplan–Meier method. Time at risk was defined as time from surgery until date of death from any cause, emigration or end of follow-up. Univariate and multivariate Cox regression analyses for OS were performed and hazard ratios were reported. The number of deaths observed was compared with the number of deaths expected, calculated by applying age and calendar year specific male mortality figures of the general Danish population and the corresponding person years of the respective cohort members. The SMR, computed as the ratio of the observed to the expected number of deaths, served as an estimate of relative risk of death, and 95% confidence intervals (CI) were computed based on the assumption that the observed number of deaths followed a Poisson distribution. The SMR were analyzed using univariate and multivariate Poisson regression models and relative risk estimates reported. Factors included in the multivariable analyses were year of surgery (< 1990, 1990–99, ≥ 2000), age at diagnosis (< 60, 60–69, ≥ 70), tumor size (≤ 2 cm, 2.1–4.9 cm, ≥ 5 cm, unknown), nodal status (negative, positive, unknown), histological type and grade (ductal grade I, II, III, unknown, other histological types), HER2 status (normal, positive, unknown), receptor status (ER, PR and AR; all negative, positive, unknown) and Ki67 (low, high, unknown). Separate models were applied substitute ER, PR, HER2 and Ki67 by subtype. A supplementary analysis including information regarding adjuvant treatment was performed. The assumption of proportional hazards was assessed by Schoenfeld residuals. All *p* values are two sided. Statistical analyses were done using SAS v9.4 (SAS Institute, Inc., Cary, USA).

## Results

The median age for the whole population of male breast cancer patients was 70 years (range 25–93 years) and more than 50% were older than 69 years (no. = 643) [6]. Further results from this entire group are presented in Appendix (Table 1 and 2a and 2b).

For the group of men included in the analysis considered primary operable (*n* = 384), we present the results in Tables 1 and 2 (listed related to decade of diagnosis respectively age at diagnosis).

**Table 1:** Histopathologic characteristics related to diagnose decade (primary operable)

Characteristics (*N* = 384)	Total	Year of operation						Test *p* value^a^
		< 1990		1990–1999		2000–		
		No	(%)	No	(%)	No	(%)	
All patients	384	59		137		188		
Lymph node status								0.001
Negative	133	13	22	41	30	79	42	
Positive	182	29	49	64	47	89	47	
Missing	69	17	29	32	23	20	11	
Tumor size (cm)								< 0.0001
≤ 2	203	23	39	69	51	111	59	
2.1–4.9	130	20	34	40	29	70	37	
5+	11	5	8	3	2	3	2	
Missing	40	11	19	25	18	4	2	
ER								0.70*
Missing	10	0	0	5	4	5	3	
< 10%	3	0	0	1	1	2	1	
≥ 10%	371	59	100	131	95	181	96	
HER2								0.36*
Missing	11	0	0	7	5	4	2	
HER2 normal	355	56	95	123	90	176	94	
HER2 positive	18	3	5	7	5	8	4	
PR								0.09*
Missing	9	0	0	5	4	4	2	
< 10%	58	9	15	28	20	21	11	
≥ 10%	317	50	85	104	76	163	87	
AR								0.46
Missing	26	3	5	10	7	13	7	
< 10%	86	18	31	32	24	36	19	
≥ 10%	272	38	64	95	69	139	74	
Ki67								< 0.0001*
Missing	12	2	4	7	5	3	1	
< 14%	241	48	81	92	67	101	54	
≥ 14%	131	9	15	38	28	84	45	
Type								0.53
IDC	351	54	92	128	93	169	90	
Other	33	5	8	9	7	19	10	
Grade								0.77
1	90	10	17	29	21	51	27	
2	178	30	51	65	47	83	44	
3	93	15	25	35	26	43	23	
Unknown	23	4	7	8	6	11	6	
Subtype								0.39*
Unknown	14	2	3	7	5	5	3	
Luminal A	194	37	63	67	49	90	48	
Luminal B	173	20	34	62	45	91	48	
HER2 enriched	0	0	0	0	0	0	0	
Triple negative	3	0	0	1	1	2	1	

*Fisher’s exact test used instead of ${\chi }^{2}$

^a^Including unknowns

**Table 2:** Histopathologic characteristics related to age at diagnosis (primary operable)

Characteristics (*N* = 384)	Total	Age at diagnosis						Test *p* value^a^
		< 60		60–69		70+		
		No	(%)	No	(%)	No	(%)	
All patients	384	96		97		191		
Lymph node status								0.007
Negative	133	39	41	36	37	58	30	
Positive	182	51	53	48	50	83	44	
Missing	69	6	6	13	13	50	26	
Tumor size (cm)								0.59
≤ 2	203	52	54	54	56	97	51	
2.1–4.9	130	30	31	29	30	71	37	
5+	11	1	1	4	4	6	3	
Missing	40	13	14	10	10	17	9	
ER								0.54*
Missing	10	3	3	3	3	4	2	
< 10%	3	0	0	2	2	1	1	
≥ 10%	371	93	97	92	95	186	97	
HER2								0.23*
Missing	11	4	4	3	3	4	2	
HER2 normal	355	84	88	92	95	179	94	
HER2 positive	18	8	8	2	2	8	4	
PR								0.96*
Missing	9	3	3	2	2	4	2	
< 10%	58	15	16	13	13	30	16	
≥ 10%	317	78	81	82	85	157	82	
AR								0.50
Missing	26	7	7	6	6	13	7	
< 10%	86	21	22	16	17	49	26	
≥ 10%	272	68	71	75	77	129	67	
Ki67								0.75*
Missing	12	4	4	4	4	4	2	
< 14%	241	58	61	59	61	124	65	
≥ 14%	131	34	35	34	35	63	33	
Type								0.11
IDC	351	91	95	84	87	176	92	
Other	33	5	5	13	13	15	8	
Grade								0.19
1	90	25	26	17	17	48	25	
2	178	34	36	52	54	92	48	
3	93	30	31	22	23	41	22	
Unknown	23	7	7	6	6	10	5	
Subtype								0.83*
Unknown	14	4	4	4	4	6	3	
Luminal A	194	47	49	49	51	98	51	
Luminal B	173	45	47	42	43	86	45	
HER2 enriched	0	0	0	0	0	0	0	
Triple negative	3	0	0	2	2	1	1	

*Fisher’s exact test used instead of ${\chi }^{2}$

^a^Including unknowns

ER was positive in 97% and PR in 83%. AR was positive in 71%.

HER2 was negative in 92%. Ki67 increased over time: 15%, 28% and 45% had ≥ 14% nuclear positivity, respectively, for the three time periods (*p* < 0.0001).

Tumor size declined statistically significantly with 39% having tumors ≤ 2 cm in the decade before 1990, and 59% after the year 2000 (*p* < 0.0001).

Lymph node status was known in 82% of all cases, of which 58% were node positive (macro- or micro-metastases).

(The same results for the group of men with tissue available (*n* = 457), data are presented in the Appendix.)

In the present cohort of early-stage MBC, 384 patients all had a mastectomy. Axillary lymph node dissection or sentinel node procedure was performed in 82% of all cases. Adjuvant treatment given is described in Table 3. 86 patients (22%) had radiotherapy, 37 (10%) chemotherapy and 182 (47%) endocrine therapy. In 243 (63%), the adjuvant treatment given was considered to be according to recommended treatment following guidelines for female breast cancer treated in the same period.

**Table 3:** Treatment in the group of primarily operable MBC (relevant treatment according to FBC guidelines)

	Frequency	Percent
Radiation		
Yes	86	28
No	217	72
Chemotherapy		
Yes	37	12
No	267	88
Endocrine therapy		
Yes	182	60
No	122	40
Tamoxifen		
Yes	168	56
No	130	44
Relevant treatment		
Yes	243	81
No	56	19
Unknown	85	

**Fig. 2:**
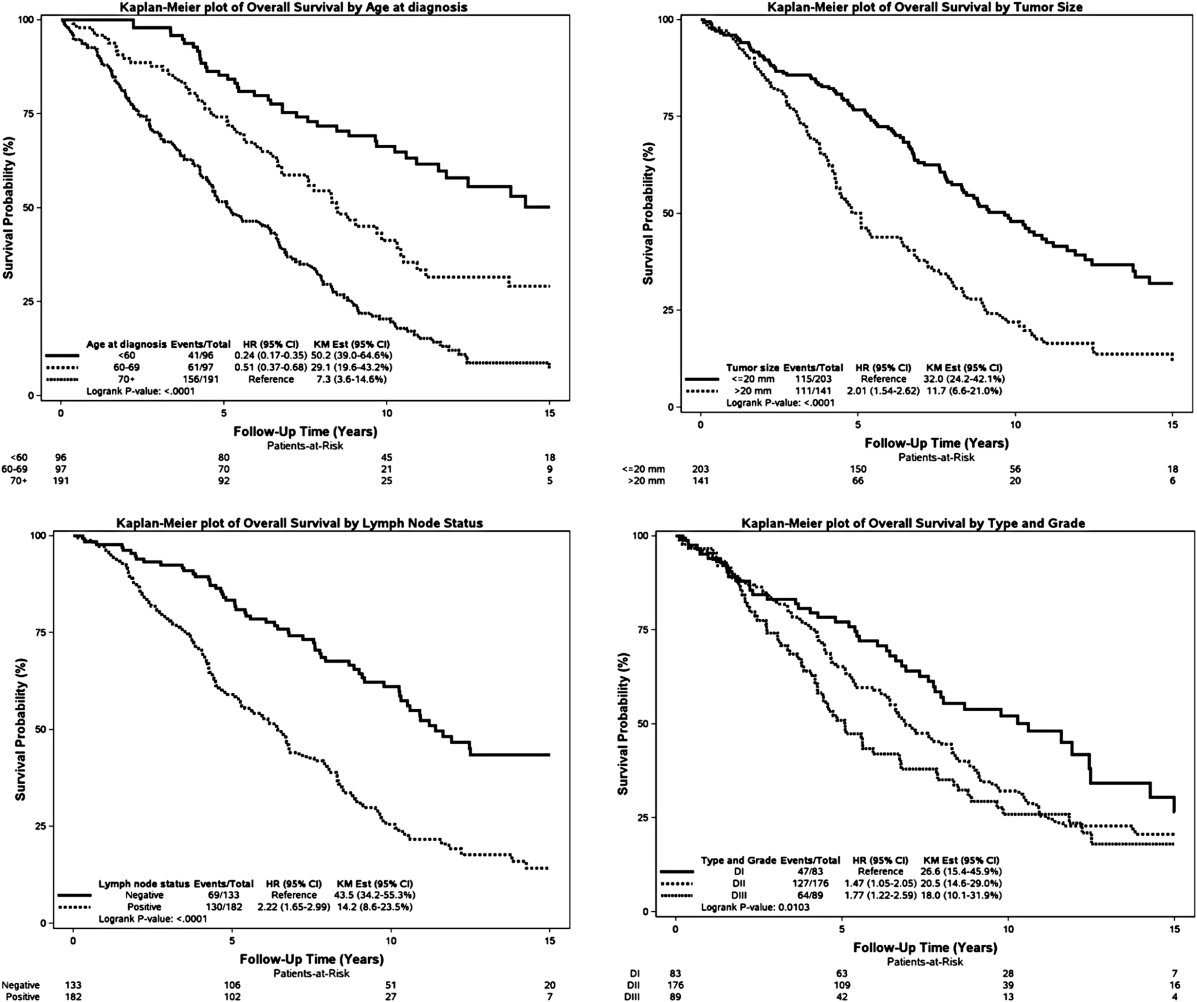

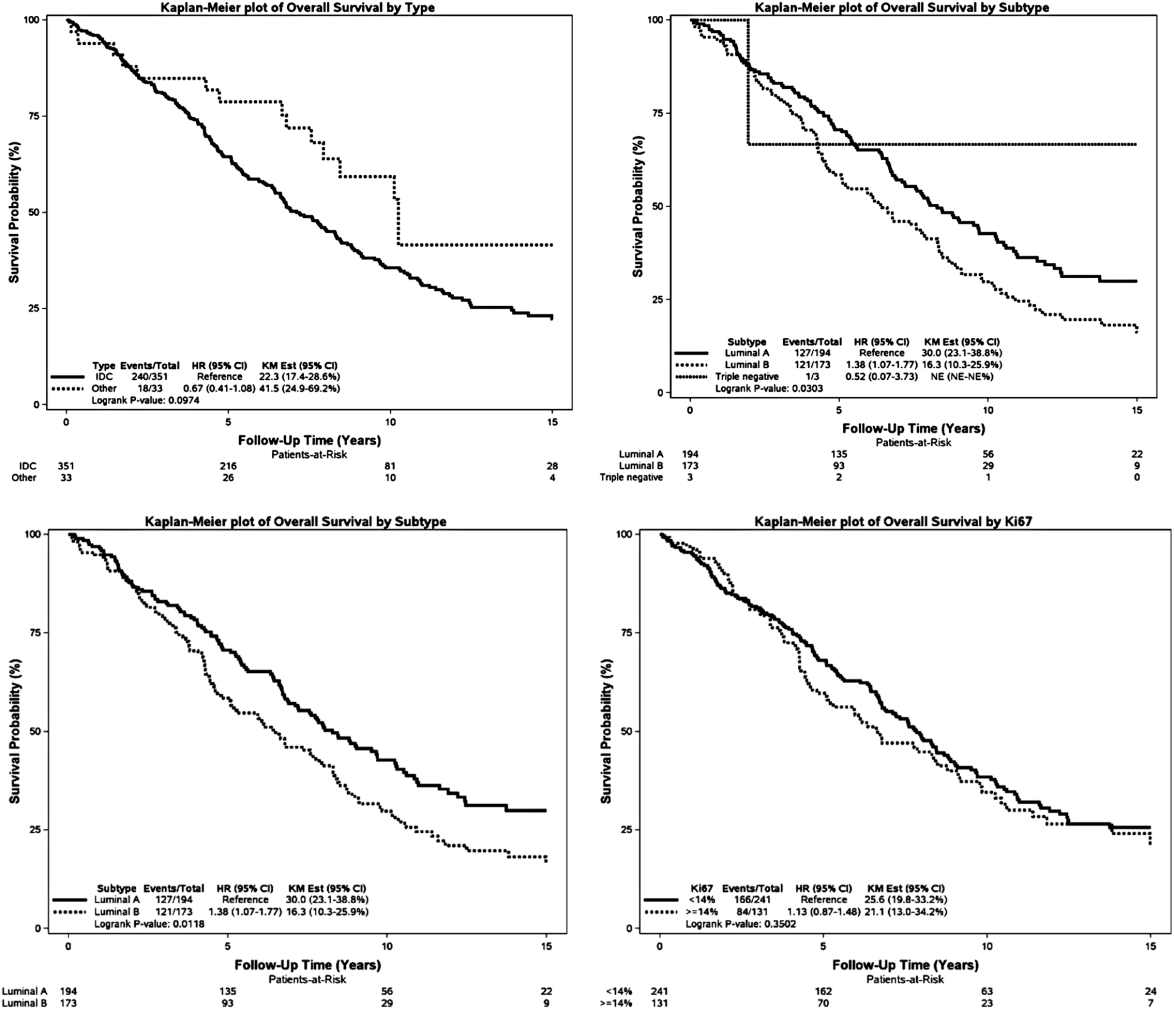

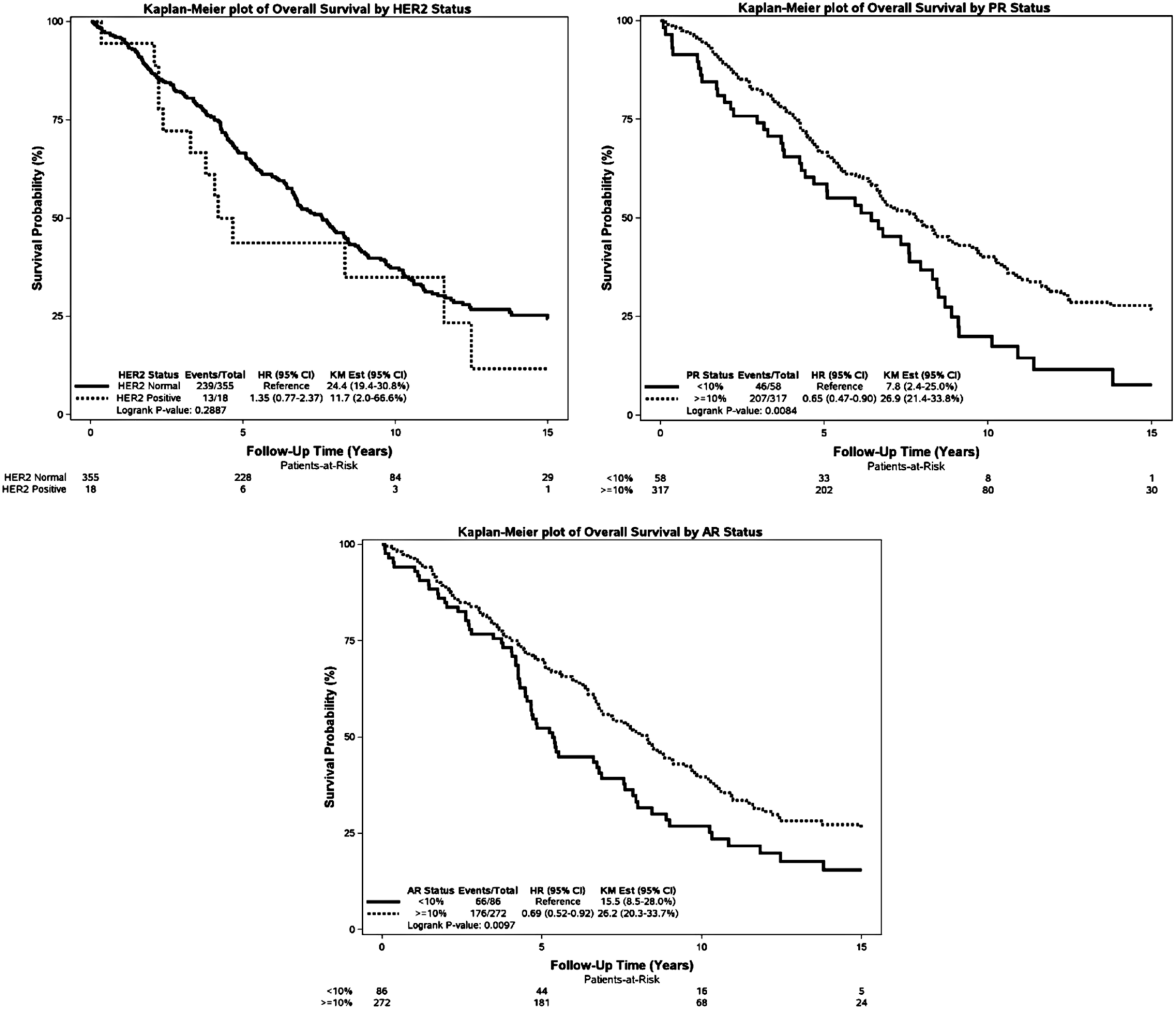
Kaplan–Meier plot for OS according to age, tumor size, lymph node status, type and grade, type, subtype (with and without triple negatives), Ki67, HER2, PR, AR

For the cohort of early-stage MBC patients, outcome as overall survival (OS), univariate, there was significant difference in OS depending on age at diagnosis, tumor size, lymph node status, tumor “type and grade” as well as subtype, PR and AR (Fig. 2).

Too few patients were ER negative to make comparison relevant.

No significant difference was demonstrated according to HER2 and KI67.

Data are presented in Fig. 2 as Kaplan–Meier plots.

OS in a multivariate model is presented in Table 4 for the following parameters: year of operation, age at diagnosis, lymph node status, tumor size, tumor type and grade, PR, AR, HER2, Ki67, as well as subtype (based on ER, PR, HER2 and Ki67).

**Table 4:** Overall survival in a multivariate model

	Overall survival		
	HR	(95% CI)	*p* value
Year of operation			0.044
< 1990	1.63	(1.11–2.38)	
1990–1999	1.22	(0.89–1.66)	
2000–	1 (ref.)		
Age at diagnosis			< 0.0001
< 60	0.22	(0.15–0.32)	
60–69	0.56	(0.41–0.76)	
70+	1 (ref.)		
Lymph node status			< 0.0001
Negative	1 (ref.)		
Positive	1.98	(1.46–2.70)	
Missing	1.97	(1.35–2.88)	
Tumor size (cm)			< 0.0001
≤ 2	1 (ref.)		
2.1–4.9	1.54	(1.17–2.04)	
5+	4.27	(2.28–8.00)	
Missing	1.43	(0.93–2.21)	
HER2			0.21
HER2 normal	1 (ref.)		
HER2 positive	1.44	(0.81–2.57)	
Missing	0.89	(0.18–4.44)	
PR			0.08
< 10%	1.39	(0.96–2.00)	
≥ 10%	1 (ref.)		
Missing	0.84	(0.15–4.89)	
AR			0.02
< 10%	1.42	(1.06–1.90)	
≥ 10%	1 (ref.)		
Missing	1.21	(0.61–2.40)	
Ki67			0.54
< 14%	1 (ref.)		
≥ 14%	1.11	(0.80–1.54)	
Missing	1.16	(0.57–2.38)	
Type			0.24
IDC	1 (ref.)		
Other	0.52	(0.17–1.54)	
Grade			0.39
1	1 (ref.)		
2	1.10	(0.78–1.56)	
3	1.34	(0.87–2.06)	
Unknown	1.28	(0.42–3.86)	

HR	(95% CI)	*p* value
Subtype		0.29
Luminal A	1 (ref.)	
Luminal B	1.24	(0.94–1.62)
Triple negative	–	–
Unknown	1.04	(0.47–2.30)

Multivariate model including year of operation, age at diagnosis, lymph node status, tumor size, HER2 status, PR status, AR status, ki67 status, type and grade and subtype. Unknowns are not included when calculating the *p* values

There were statistically significant associations between OS and age at diagnosis, lymph node status, tumor size and AR status.

When looking at SMR in a univariate model, significant associations were found for year of operation, age at diagnosis, tumor size, type and grade as well as PR, HER2, Ki67, lymph node involvement and subtype. No association was found between SMR and AR. Data are presented in Table 5.

**Table 5:** The effect of patient and tumor characteristics on standardized mortality ratio (SMR) evaluated with crude estimates and with estimates of relative risk in univariate and multivariate Poisson models, including year of surgery, age at diagnosis, lymph node status, tumor size, HER2, PR, AR, Ki67, histological type and grade

	Number of deaths		Crude		Relative risk estimates					
					Univariate			Multivariate		
	Observed	Expected	SMR (95% CI)		RR (95% CI)		*p* value	RR (95% CI)		*p* value
Total	258	160.6	1.61	(1.42–1.81)						
Year of operation							0.02			0.33
< 1990	56	27.7	2.02	(1.56–2.63)	1.65	(1.17–2.33)		1.33	(0.91–1.96)	
1990–1999	115	67.4	1.71	(1.42–2.05)	1.32	(0.99–1.75)		1.08	(0.79–1.47)	
2000–	87	65.5	1.33	(1.07–1.64)	1 (ref.)			1 (ref)		
Age at diagnosis							< 0.0001			0.0001
< 60	41	12.8	3.20	(2.36–4.34)	2.76	(1.93–3.95)		2.18	(1.47–3.22)	
60–69	61	29.7	2.05	(1.60–2.64)	1.72	(1.27–2.33)		1.71	(1.23–2.37)	
70+	156	118.1	1.32	(1.13–1.55)	1 (ref.)			1 (ref)		
Lymph node status							< 0.0001			< 0.0001
Negative	69	64.5	1.07	(0.85–1.36)	1 (ref.)			1 (ref)		
Positive	130	50.7	2.57	(2.16–3.05)	2.57	(1.90–3.48)		1.90	(1.37–2.63)	
Missing	59	45.5	1.30	(1.00–1.67)	1.24	(0.87–1.76)		1.39	0.96–2.01)	
Tumor size (cm)							< 0.0001			0.002
≤ 2	115	92.6	1.24	(1.03–1.49)	1 (ref.)			1 (ref)		
2.1–4.9	100	51.6	1.94	(1.59–2.36)	1.59	(1.21–2.08)		1.25	(0.94–1.68)	
5+	11	2.0	5.58	(3.09–10.08)	4.89	(2.61–9.16)		3.72	(1.91–7.23)	
Missing	32	14.4	2.22	(1.57–3.14)	1.81	(1.22–2.68)		1.42	(0.93–2.17)	
HER2							0.01			0.11
HER2 normal	239	153.4	1.56	(1.37–1.77)	1 (ref.)			1 (ref)		
HER2 positive	13	3.7	3.48	(2.02–6.00)	2.27	(1.30–3.98)		1.66	(0.92–2.97)	
Missing	6	3.5	1.71	(0.77–3.81)	1.12	(0.49–2.51)		1.01	(0.20–5.02)	
PR							0.0002			0.002
< 10%	46	16.4	2.81	(2.11–3.75)	1.92	(1.39–2.64)		1.74	(1.24–2.45)	
≥ 10%	207	141.0	1.47	(1.28–1.68)	1 (ref.)			1 (ref)		
Missing	5	3.3	1.52	(0.63–3.65)	1.04	(0.43–2.53)		0.89	(0.13–5.95)	
AR							0.22			0.09
< 10%	66	36.3	1.82	(1.43–2.32)	1.20	(0.90–1.59)		1.32	(0.96–1.81)	
≥ 10%	176	115.0	1.53	(1.32–1.77)	1 (ref.)			1 (ref)		
Missing	16	9.4	1.70	(1.04–2.78)	1.15	(0.69–1.92)		1.22	(0.62–2.40)	
Ki67							0.03			0.20
< 14%	166	113.6	1.46	(1.26–1.70)	1 (ref.)			1 (ref)		
≥ 14%	84	43.1	1.95	(1.57–2.41)	1.35	(1.04–1.76)		1.22	(0.90–1.66)	
Missing	8	3.9	2.03	(1.01–4.05)	1.40	(0.69–2.85)		1.46	(0.57–3.76)	
Type							0.005			0.10
IDC	240	141.0	1.70	(1.50–1.93)	1 (ref.)			1 (ref)		
Other	18	19.6	0.92	(0.58–1.46)	0.53	(0.33–0.86)		0.52	(0.23–1.16)	
Grade							< 0.0001			0.26
1	50	44.3	1.13	(0.85–1.49)	1 (ref.)			1 (ref)		
2	128	75.3	1.70	(1.43–2.02)	1.57	(1.13–2.18)		1.19	(0.84–1.68)	
3	66	24.8	2.66	(2.09–3.38)	2.46	(1.70–3.57)		1.41	(0.93–2.15)	
Unknown	14	16.2	0.87	(0.51–1.46)	0.78	(0.43–1.42)		1.30	(0.52–3.27)	
Subtype							0.0001			0,02
Luminal A	127	98.5	1.29	(1.08–1.53)	1 (ref.)			1(ref.)		
Luminal B	121	55.7	2.17	(1.82–2.60)	1.72	(1.33–2.21)		1.45	(1.10–1.92)	
Triple negative	1	1.2	0.86	(0.12–6.11)	–	–		–	–	
Unknown	9	5.3	1.69	(0.88–3.25)	1.29	(0.66–2.55)		1.43	(0.62–3.31)	

A separate model was applied substituting ER, PR, HER2 and Ki67 by subtype. *p* values do not include categories with missing values

In the multivariate model for SMR, significant associations were only found for age at diagnosis, lymph node status, tumor size, PR, and Luminal A and B subtype.

## Discussion

This study represents a large Danish cohort of male breast cancer diagnosed over a period of 3 decades.

We have previously presented the clinical data [6] and here, we present the related clinicopathological characteristics.

MBC was dominated by tumors of ductal type and mostly grade 2. They were almost exclusively ER positive and of luminal subtypes. All HER2-positive cancers were ER positive, too.

The main strength of this study is that it is based on a national population cohort and that nation-wide survival data were available for a period of 30 years. The Danish healthcare system is tax-supported, free of charge and available to everybody. This system is optimal for national population-based studies, as it gives us a very precise picture of the diagnosis and treatment of all Danish breast cancer patients during the study period. These facts allow for analyses based on data free of selection bias.

The high-quality Danish registers include Statistics Denmark allowing for high quality of SMR analyses. SMR compensates for not having breast cancer specific mortality data.

All available tumor tissue blocks were independently re-classified by three experienced breast cancer pathologists to avoid inter-observer variability assuring quality of the data.

Limitations of the study are the small number of male breast cancer patients, requiring a long study period to include enough patients for statistics to make sense, and, as for most other retrospective studies including more than 30-year-old data, the missing variables especially among the oldest data. The quality of the oldest tissue blocks from the first decade was not as good as the tissue blocks from the last decade. This might have affected the estimation of tumor characteristics.

Our findings are in agreement with several recent studies [11, 12, 19].

Our results confirm that AR seems to play a role in MBC. This is of special interest, as AR positivity is being studied in ER-positive disease [20]. This receptor could eventually play role in treatment, as it seems to be a new possible treatment target and thereby both a prognostic and predictive marker [4, 21, 22].

The fact that only 63% got the recommended treatment for female breast cancer (FBC) could reflect the group’s compliance that they are men, that they are older and that anti-hormone treatment probably has even more side-effects for men than for FBC patients, or that their tolerance is lower.

Studies have shown that both OS and disease-free survival (DFS) was significantly affected by low adherence [23, 24]

When looking at standardized mortality rates (SMR) in a univariate model, significant associations were found for year of operation, age at diagnosis, tumor size, type and grade as well as PR, HER2, Ki67, lymph node involvement and subtype.

This is concordant with SMR for FBC [6], and they are well-known prognostic factors important for the indication of adjuvant treatment.

The overwhelming amount of Luminal subtype is in accordance with former published studies [11, 25].

In other studies, Luminal A subtype based on immunohistochemical parameters seems to be the more dominant [13] and is overall also in concord with the present study. However, our study shows that the subtype tends to change from Luminal A towards Luminal B in the later (more recent) decade. The more prominent occurrence of Luminal B in comparison to what is found in female cohorts was also described in a review article from Giordano [25] and in the studies from Cardoso or Vermeulen on EORTC material [11, 19]. Their data are from men diagnosed from 1990 to 2010, thus the two most recent of our decades. The change in our observation concerning the luminal subtypes might reflect the observed change in Ki67,

It has been shown that Ki67 antigenicity is lost more rapidly than other targets of immune stains [26]. This could explain our findings with a higher Ki67 in the last decades compared to the previous (15–28–45%) and is, therefore, in our opinion, most obviously not a result of changes in the biology of MBC over time.

In addition, there is a well-known inter-laboratory variation in Ki67 evaluation [27], which we have tried to avoid, by three pathologists retesting and evaluating the results together. These findings should be taken into consideration when interpreting distribution of intrinsic subtypes, as Ki67 ratio cut-off is used in subtyping into Luminal A and Luminal B types.

This can, therefore, be crucial when doing immunohistochemical subtyping, especially in older material.

A smaller study of 67 MBC from Sanches-Muñjos, doing PAM50 subtyping based on a 50-gene signature also showed an overweight of Luminal B subtype [15]. This means that we will have to be aware that more men are Luminal B, compared to females with luminal type breast cancer [28].

We found a significantly better SMR for Luminal A subtype than for Luminal B subtype, also well known from female breast cancer [29, 30]. This could indicate the importance of doing molecular subtyping for MBC to distinguish between those patients who will benefit from chemotherapy and those, who will not, if it can be calculated as for postmenopausal FBC.

The small number of MBC means that this will be an affordable task.

It is our intention to do PAM50 on a part of this material as well as tumor-infiltrating lymphocytes (TILS) and BRCA testing in the hope of further characterizing the population.

A better knowledge of this area might contribute to optimizing treatment of MBC and thereby improving the prognosis.

## Electronic supplementary material

Below is the link to the electronic supplementary material.

Supplementary file1 (DOCX 46 kb)
