# Supplementary material for: Male breast cancer: clinicopathological characterization of a National Danish cohort 1980–2009
Source: Breast Cancer. 2020 Feb 27;27(4):683–95. doi: 10.1007/s12282-020-01066-3 (PMC7297815; doi:10.1007/s12282-020-01066-3)
Supplement: Supplementary file 1 — Supplementary file1 (DOCX 46 kb) [file 12282_2020_1066_MOESM1_ESM.docx]

“Male Breast Cancer. Clinicopathological characterization of a national Danish cohort 1980-2009”

Appendix

Corresponding author: MD, associate professor Anne Marie Bak Jylling.

[Anne.marie.bak.jylling@rsyd.dk](mailto:Anne.marie.bak.jylling@rsyd.dk). Phone: +45 26 82 35 75.

ORCID id: 0000-0002-8071-549X

APPENDIX

The median age for the whole population of male breast cancer patients was 70 years (range 25-93 years) and more than and more than 50% were older than 69 years (no.= 643) (6).

In our study almost all MBC were of luminal subtype with only very few showing HER2 overexpression/amplification and even fewer were triple negative. The Most predominant histological type was invasive ductal carcinoma (91%). The remainder included lobular, cribriform, papillary, mucinous, tubular, micropapillary, and clearcell carcinomas. Most tumors were malignancy grade II (46%).

Males had more often ER and PR positive and HER2 negative disease than females.

Ki67 ratio increased over the period of study and was significantly lower for the early calendar periods.

AR was also positive in a majority.

When lymph node status was known more than half had metastatic spread.

In our study almost all MBC were of luminal subtype with only very few showing HER2 overekspression/amplification and even fewer were triple negative. They had higher histological grade and larger size than tumors of other subtypes.

The most common subtype was luminal A followed by luminal B. The proportion of Luminal B though raised over time so at the latest period (>2000) there was an almost equal distribution of Luminal A and Luminal B tumor subtypes.

23 patients had locally advanced or disseminated disease at the initial diagnosis.

Number of missing data reduced over the time period.

Data are presented in Table 1.

For the group of all men with tissue available (n= 457) ER was positive in 94% and PR in 80%. AR was positive in 66%.

HER2 was negative in 91%. KI67 increased over time (respectively 16%, 31% and 45% had > 14% positive nuclear staining grouped by the decades (<1990, 1990-2000, 2000>), which is statistically significant (P<0,0001).

Tumor size declined over time with 31% having tumor size < 2 cm <1990, and 58% < 2 cm in 2000>, which is statistically significant (p < 0,0001).

Lymph node status was known in 75% of cases by axillary lymph node dissection or sentinel node procedure. 59% had metastatic disease to the lymph nodes (macro- or micro-metastases). Difference in lymph node status was the only with significant difference between age groups (p=0,005)

All data are presented in Table 2 a and 2 b. with respect to year of operation and age at diagnosis.

| Characteristics  (N=643) | |  | +/- Tissue | | | | Test p-value^#^ |
| --- | --- | --- | --- | --- | --- | --- | --- |
|  |  |  | + Tissue | | - Tissue | |  |
|  |  | Total | No. | (%) | No. | (%) |  |
| All patients | |  | 457 |  | 186 |  |  |
| Year of operation | |  |  |  |  |  | <0.0001 |
|  | <1990 | 183 | 88 | 19 | 95 | 51 |  |
|  | 1990-1999 | 222 | 171 | 37 | 51 | 27 |  |
|  | 2000- | 238 | 198 | 44 | 40 | 22 |  |
| Age at diagnosis | |  |  |  |  |  | 0.31 |
|  | <60 | 148 | 112 | 24 | 36 | 19 |  |
|  | 60-69 | 164 | 118 | 26 | 46 | 25 |  |
|  | 70+ | 331 | 227 | 50 | 104 | 56 |  |
| Lymph node status | |  |  |  |  |  | <0.0001 |
|  | Negative | 167 | 139 | 30 | 28 | 15 |  |
|  | Positive | 232 | 203 | 44 | 29 | 16 |  |
|  | ? | 244 | 115 | 25 | 129 | 69 |  |
| Tumor size (cm) | |  |  |  |  |  | <0.0001 |
|  | ≤2 | 254 | 218 | 48 | 36 | 19 |  |
|  | 2.1-4.9 | 174 | 154 | 34 | 20 | 11 |  |
|  | 5+ | 18 | 16 | 3 | 2 | 1 |  |
|  | missing | 197 | 69 | 15 | 128 | 69 |  |

Table 1. Patient characteristics. All patients. All percentages are column percentages. # including unknowns.

| Characteristics  (N=457) | |  | Year of operation | | | | | | Test p-value^#^ |
| --- | --- | --- | --- | --- | --- | --- | --- | --- | --- |
|  |  |  | <1990 | | 1990-1999 | | 2000- | |  |
|  |  | Total | No. | (%) | No. | (%) | No. | (%) |  |
| All patients | | 457 | 88 |  | 171 |  | 198 |  |  |
| Lymph node status | |  |  |  |  |  |  |  | <0.0001 |
|  | Negative | 139 | 14 | 16 | 44 | 26 | 81 | 41 |  |
|  | Positive | 203 | 36 | 41 | 74 | 43 | 93 | 47 |  |
|  | Missing | 115 | 38 | 43 | 53 | 31 | 24 | 12 |  |
| Tumor size (cm) | |  |  |  |  |  |  |  | <0.0001 |
|  | ≤2 | 218 | 27 | 31 | 77 | 45 | 114 | 58 |  |
|  | 2.1-4.9 | 154 | 23 | 26 | 55 | 32 | 76 | 38 |  |
|  | 5+ | 16 | 7 | 8 | 5 | 3 | 4 | 2 |  |
|  | Missing | 69 | 31 | 35 | 34 | 20 | 4 | 2 |  |
| ER | |  |  |  |  |  |  |  | 0.80* |
|  | Missing | 12 | 1 | 1 | 5 | 3 | 6 | 3 |  |
|  | <10% | 14 | 4 | 5 | 5 | 3 | 5 | 3 |  |
|  | ≥10% | 431 | 83 | 94 | 161 | 94 | 187 | 94 |  |
| HER2 | |  |  |  |  |  |  |  | 0.32 |
|  | Missing | 14 | 1 | 1 | 8 | 5 | 5 | 3 |  |
|  | HER2 Normal | 414 | 83 | 94 | 149 | 87 | 182 | 92 |  |
|  | HER2 Positive | 29 | 4 | 5 | 14 | 8 | 11 | 6 |  |
| PR | |  |  |  |  |  |  |  | 0.08* |
|  | Missing | 12 | 1 | 1 | 6 | 3 | 5 | 2 |  |
|  | <10% | 79 | 15 | 17 | 39 | 23 | 25 | 13 |  |
|  | ≥10% | 366 | 72 | 82 | 126 | 74 | 168 | 85 |  |
| AR | |  |  |  |  |  |  |  | 0.046 |
|  | Missing | 35 | 10 | 11 | 11 | 7 | 14 | 7 |  |
|  | <10% | 119 | 29 | 33 | 50 | 29 | 40 | 20 |  |
|  | ≥10% | 303 | 49 | 56 | 110 | 64 | 144 | 73 |  |
| Ki67 | |  |  |  |  |  |  |  | <0.0001 |
|  | Missing | 15 | 3 | 3 | 8 | 5 | 4 | 2 |  |
|  | <14% | 285 | 71 | 81 | 110 | 64 | 104 | 53 |  |
|  | ≥14% | 157 | 14 | 16 | 53 | 31 | 90 | 45 |  |
| Type | |  |  |  |  |  |  |  | 0.52 |
|  | IDC | 419 | 80 | 91 | 160 | 94 | 179 | 90 |  |
|  | Other | 38 | 8 | 9 | 11 | 6 | 19 | 10 |  |
| Grade | |  |  |  |  |  |  |  | 0.77 |
|  | 1 | 102 | 19 | 22 | 32 | 19 | 51 | 26 |  |
|  | 2 | 209 | 42 | 48 | 79 | 46 | 88 | 44 |  |
|  | 3 | 121 | 23 | 26 | 50 | 29 | 48 | 24 |  |
|  | Unknown | 25 | 4 | 5 | 10 | 6 | 11 | 6 |  |
| Subtype | |  |  |  |  |  |  |  | 0.03* |
|  | Unknown | 17 | 3 | 3 | 8 | 5 | 6 | 3 |  |
|  | Luminal A | 227 | 56 | 64 | 78 | 45 | 93 | 47 |  |
|  | Luminal B | 201 | 25 | 28 | 82 | 48 | 94 | 47 |  |
|  | HER2 Enriched | 0 | 0 | 0 | 0 | 0 | 0 | 0 |  |
|  | Triple negative | 12 | 4 | 5 | 3 | 2 | 5 | 3 |  |

Table 2.a Histopathologic characteristics related to decade of operation (all men with tissue). *: Fisher’s exact test used instead of $\chi^{2}$; #: Including unknowns

| Characteristics  (N=457) | |  | Age at diagnosis | | | | | | Test p-value^#^ |
| --- | --- | --- | --- | --- | --- | --- | --- | --- | --- |
|  |  |  | <60 | | 60-69 | | 70+ | |  |
|  |  | Total | No. | (%) | No. | (%) | No. | (%) |  |
| All patients | | 457 | 112 |  | 118 |  | 227 |  |  |
| Lymph node status | |  |  |  |  |  |  |  | 0.005 |
|  | Negative | 139 | 40 | 36 | 39 | 33 | 60 | 26 |  |
|  | Positive | 203 | 56 | 50 | 54 | 46 | 93 | 41 |  |
|  | Missing | 115 | 16 | 14 | 25 | 21 | 74 | 33 |  |
| Tumor size (cm) | |  |  |  |  |  |  |  | 0.72 |
|  | ≤2 | 218 | 56 | 50 | 58 | 49 | 104 | 46 |  |
|  | 2.1-4.9 | 154 | 34 | 30 | 35 | 30 | 85 | 37 |  |
|  | 5+ | 16 | 3 | 3 | 5 | 4 | 8 | 4 |  |
|  | Missing | 69 | 19 | 17 | 20 | 17 | 30 | 13 |  |
| ER | |  |  |  |  |  |  |  | 0.36* |
|  | Missing | 12 | 5 | 4 | 3 | 3 | 4 | 2 |  |
|  | <10% | 14 | 2 | 2 | 6 | 5 | 6 | 3 |  |
|  | ≥10% | 431 | 105 | 94 | 109 | 92 | 217 | 95 |  |
| HER2 | |  |  |  |  |  |  |  | 0.13* |
|  | Missing | 14 | 7 | 6 | 3 | 3 | 4 | 2 |  |
|  | HER2 Normal | 414 | 95 | 85 | 110 | 93 | 209 | 92 |  |
|  | HER2 Positive | 29 | 10 | 9 | 5 | 4 | 14 | 6 |  |
| PR | |  |  |  |  |  |  |  | 0.38* |
|  | Missing | 12 | 6 | 5 | 2 | 2 | 4 | 2 |  |
|  | <10% | 79 | 21 | 19 | 20 | 17 | 38 | 17 |  |
|  | ≥10% | 366 | 85 | 76 | 96 | 81 | 185 | 81 |  |
| AR | |  |  |  |  |  |  |  | 0.30 |
|  | Missing | 35 | 12 | 11 | 9 | 8 | 14 | 6 |  |
|  | <10% | 119 | 27 | 24 | 25 | 21 | 67 | 30 |  |
|  | ≥10% | 303 | 73 | 65 | 84 | 71 | 146 | 64 |  |
| Ki67 | |  |  |  |  |  |  |  | 0.27* |
|  | Missing | 15 | 7 | 6 | 4 | 3 | 4 | 2 |  |
|  | <14% | 285 | 65 | 58 | 76 | 64 | 144 | 63 |  |
|  | ≥14% | 157 | 40 | 36 | 38 | 32 | 79 | 35 |  |
| Type | |  |  |  |  |  |  |  | 0.11 |
|  | IDC | 419 | 106 | 95 | 103 | 87 | 210 | 93 |  |
|  | Other | 38 | 6 | 5 | 15 | 13 | 17 | 7 |  |
| Grad | |  |  |  |  |  |  |  | 0.36 |
|  | 1 | 102 | 26 | 23 | 24 | 20 | 52 | 23 |  |
|  | 2 | 209 | 41 | 37 | 57 | 48 | 111 | 49 |  |
|  | 3 | 121 | 38 | 34 | 31 | 26 | 52 | 23 |  |
|  | Unknown | 25 | 7 | 6 | 6 | 5 | 12 | 5 |  |
| Subtype | |  |  |  |  |  |  |  | 0.60* |
|  | Unknown | 17 | 7 | 6 | 4 | 4 | 6 | 3 |  |
|  | Luminal A | 227 | 52 | 46 | 59 | 50 | 116 | 51 |  |
|  | Luminal B | 201 | 51 | 46 | 50 | 42 | 100 | 44 |  |
|  | HER2 Enriched | 0 | 0 | 0 | 0 | 0 | 0 | 0 |  |
|  | Triple negative | 12 | 2 | 2 | 5 | 4 | 5 | 2 |  |

Table 2.b. Histopathologic characteristics related to age at diagnosis (all men with tissue). *: Fisher’s exact test used instead of $\chi^{2}$; #: Including unknowns
